# Re-analyzing and confirming a differential use of redintegration in students with mild and borderline intellectual disabilities

**DOI:** 10.3389/fpsyg.2024.1278458

**Published:** 2024-08-22

**Authors:** Gunnar Bruns

**Affiliations:** Rehabilitation Sciences on Special Learning Needs, Institute of Educational Research, School of Education, University of Wuppertal, Wuppertal, Germany

**Keywords:** working memory, mild intellectual disability (MID), borderline intellectual disability, redintegration, developmental trajectories psychometrika

## Abstract

While numerous studies on verbal working memory have investigated the capacity of the phonological loop and the effectiveness of rehearsal as one core process for maintaining the memory trace, the reconstruction of the memory trace from long-term memory, called redintegration, has been studied less thoroughly. This holds particularly for the population of students with special educational learning needs and mild and borderline intellectual disabilities (MBID). In a previous study, we found a differential developmental relation between the effectiveness of redintegration and vocabulary size, counter-intuitively suggesting that students with MBID tend to show less effective redintegration with higher vocabulary size. However, differential item functioning (DIF) in the picture naming task may have biased the result. Therefore, the current study is a re-analysis of this interaction controlling for DIF in the vocabulary measure. To this end, the items of the picture naming task (*k* = 95) were analyzed through a Rasch model, and *k* = 29 biased items were excluded. The resulting corrected vocabulary score was used to predict the redintegration effectiveness, comparing students with and without MBID. The interaction remains significant, supporting the original finding that students with MBID have a differential developmental pattern and are less able to make adequate use of a growing vocabulary when reconstructing traces in their working memory. Implications of this result for the understanding of MBID and further research directions are discussed.

## 1 Introduction

This brief research report seeks to re-analyze and hopes to confirm an unexpected group interaction effect found in a previous developmental trajectory study (Bruns et al., 2019). However, differential item functioning (DIF) can be discussed as a possible methodological caveat. Therefore, DIF needs to be excluded to re-analyze the developmental trajectory interaction.

Compared to students with specific learning disabilities, children with mild and borderline intellectual disabilities (MBID), which correspond to the population of German schools for special learning needs, have not been the focus of research. Apart from distinct syndromes such as Down syndrome or Williams syndrome, the causes and description of cognitive prerequisites of MBID remain unclear and underspecified. Mostly, the phenomenon is described rather superficially and without precise assumptions of causal factors (e.g., Schröder, 2000; Kretschmann, 2007; Shaw, 2010 as cited in Hassiotis, 2015). Some evidence suggests that cognitive processes, particularly phonological and visuo-spatial working memory, are an important prerequisite, where children with MBID show deficits (Hasselhorn and Mähler, 2007; Mähler, 2007; Cornoldi and Giofrè, 2014; van der Molen et al., 2014; Lifshitz et al., 2016). While most of these studies have focused on verbal working memory capacity and rehearsal as a retention process in the phonological loop (Baddeley, 1986), none of them considered redintegration. This cognitive process is at the transition between working and long-term memory and can be conceptualized as the reconstruction of degraded working memory traces from the mental lexicon (Schweickert, 1993). The study of Bruns et al. (2019) was the first to investigate redintegration in students with MBID.

In this study, a developmental trajectory approach (DT, Thomas et al., 2009) was employed to investigate developmental relations between working memory and several predictors, and compare the relations between children with and without MBID. When viewed in relation to chronological age and cognitive capacity, the effectiveness of the redintegration process seemed unimpaired at first glance. In contrast, the DT analysis yielded a rather surprising group interaction specifically when redintegration was set in relation to vocabulary size, which was measured by a picture naming task as a proxy for the mental lexicon. The interaction was interpreted as a differential developmental pattern: For students with MBID, higher vocabulary scores were associated with *less* effective redintegration. The more words a child with MBID had in the mental lexicon, the lower was the average redintegration effectiveness, meaning that it presumably had greater difficulties in reconstructing working memory items from long-term memory.

However, before this effect can be reliably interpreted, a lack of measurement invariance on item level needs to be excluded as a possible reason for group differences. For dichotomous items of performance tests, the probabilistic Rasch model (Fischer and Molenaar, 1995) is a useful framework to assess the measurement invariance across different groups. If items are biased, this leads to different parameter estimates per group, called differential item functioning (DIF, Glas and Verhelst, 1995), indicating that they are systematically easier or harder to solve for certain sub-populations. DIF can be tested through a likelihood ratio test (LR-Test, Andersen, 1973), or for small sample sizes, Koller et al. (2012) suggest non-parametric quasi-exact tests based on a large number of MCMC simulated matrices (Ponocny, 2001; Verhelst, 2008). The statistic *T*_10_ can be interpreted as a non-parametric equivalent of the LR-Test (Koller et al., 2012), providing an indicator if subgroup invariance is violated on the scale level. The discrepancy can also be analyzed for each item separately to identify and exclude biased items. This procedure is described in more detail in the Methods section.

For the DT method as research design, the reader is referred to the original study (Bruns et al., 2019) and the detailed explanations by Thomas et al. (2009). One particular advantage of DTs is their capability to depict *developmental relations*, as DTs “aim […] to construct a function *linking performance with age* on a specific experimental task and then to assess whether this function differs between the typically developing group and the disorder group” (Thomas et al., 2009, p. 336). For each group, a regression line is fitted, which can be compared regarding intercept and slope coefficients between groups, tasks, and their interactions. In accordance with the original study, the current study differentiates three possible linear scenarios that can result from this analysis: (a) *delayed onset* can be observed when the groups differ at the intercept, that is, the onset of development; (b) *slowed rate* manifests a difference in the slopes; and (c) a combination of delayed onset and slowed rate.

Summing up, to exclude DIF as possible cause for the differential pattern, the following research questions and hypotheses are derived: (1) are there subscales and items in the vocabulary task that have a substantial measurement bias? It is plausible to expect that at least a few items are systematically harder or easier to solve for children with MBID (i.e., show DIF). (2) Subsequently, does the differential developmental pattern for the redintegration process hold when a corrected vocabulary score is used as predictor? If this interaction fails to become significant, DIF will have to be considered as (one) causal factor for the interaction, and hence further interpretation of the interaction will not be possible. On the other hand, if the interaction remains significant, DIF is rather unlikely to be the reason, allowing other interpretations that children with MBID have a less effective reconstruction mechanism when accessing items from long-term memory.

## 2 Methods

### 2.1 Participants

The sample consisted of *N* = 207 German students, analogous to the sample in the original study (Bruns et al., 2019): 93 belonged to the group of MBID (53 male students, *M* = 13;2 years, *SD* = 2;3, range 7;4–17;1 years). The TD group consisted of 114 students without learning difficulties that were matched for mental age (50 male students, *M* = 8;9 years, *SD* = 1;1, range 6;0–13;5 years). The sample characteristics are shown in Table 1 in the Results section. There were no significant differences between the groups regarding gender (*p* = 0.082), cognitive capacity raw scores (*p* = 0.899), and overall vocabulary size raw scores (*p* = 0.599). The significant differences regarding age (*p* < 0.001) and intelligence (*p* < 0.001) are intended by design and result from the mental age matching approach (Zigler and Balla, 1982).

**Table 1:** Sample characteristics: means and standard deviations for background variables for students with MBID and the TD control group.

	MBID (*n* = 93)		TD (*n* = 114)		Significance
	*M*	*SD*	*M*	*SD*	
Gender (M/F)	53/40		50/64		χ^2^(1) = 3.026; *p* = 0.082; φ = 0.12
Age (Years; months)	13;2^a^	2;3	8;9	1;1	*t*(119.92) = 20.805; *p* = 0.001; *d* = 3.16
Cognitive capacity (CFT 1-R raw scores)	65.8^b^	11.8	65.6	12.9	*t*(187.04) = 0.127; *p* = 0.899; *d* = 0.02
Vocabulary (WWT 6–10 raw scores)	53.4	16.8	54.6	14.1	*t*(179.89) = 0.526; *p* = 0.599; *d* = 0.07
Intelligence (IQ norm scores)	76.0^a^	8.4	105.4	9.9	*t*(200.92) = 22.869; *p* = 0.001; *d* = 3.16

To measure cognitive capacity, the raw scores of the CFT 1-R (Weiß and Osterland, 2012) were used. The raw scores of the expressive picture naming task WWT 6–10 (Glück, 2011) were used to measure the vocabulary size. As the groups are matched for mental age (i.e., cognitive capacity and absolute vocabulary size), the differences in age and IQ are intended. Due to unequal variances, the corrected degrees of freedom are reported for the *t*-tests.^a^*n* = 90. ^b^*n* = 84.

To qualify for the MBID group, students had to meet the following criteria: a formal diagnosis of special learning needs; an IQ below 85 as measured during the formal special needs assessment; no other developmental disorders, such as ADHD and specific learning disabilities, according to teacher report. Students with MBID were recruited from special educational needs schools in an urban environment in Germany. Students of the TD group attended regular primary schools and had no diagnosis of special educational needs or developmental disorders. In addition, their IQ and vocabulary scores had to be at least average, that is, IQ > 85 measured by the CFT 1-R (Weiß and Osterland, 2012) and a vocabulary T-Score > 40 measured by the WWT 6–10 (Glück, 2011; as described below).

As in the original study, ethics approval was not required according to the guidelines of the state NRW in Germany, and data collection was carried out following the recommendations of the Federation of German Psychologist Association that written informed consent be obtained from all subjects’ parents or caregivers in accordance with the Declaration of Helsinki. All participants gave their oral agreement to participate, and their parents were informed of the objectives of the study, the nature of the tasks to be administered, and that they could withdraw their agreement at any time. Permission to conduct tests in schools was obtained from the school principals.

### 2.2 Procedure and materials

Test materials were equivalent to those in the original study, where they are described in more detail (Bruns et al., 2019). Since only redintegration and vocabulary are relevant to the research question, they are only briefly introduced here: (1) word-span repetition tasks with real words and pseudowords and (2) an expressive vocabulary picture naming task.

#### 2.2.1 Redintegration: word-span tasks

To measure the effectiveness of the redintegration process, the lexicality effect in word-span tasks was investigated. We used four different word-span tasks in 2 (Length: short vs. long) × 2 (Lexicality: real vs. pseudo) conditions, for example, short real word-span: “Haus–Stern–Schuh” (house–star–shoe), or long pseudoword-span: “karflumen–franulich–wuralten”. The stimuli were taken from the AGTB 5–12 (Hasselhorn et al., 2012) and from Hasselhorn et al. (2010), for a full list see in Table A-1 in Bruns et al. (2019). Children were instructed to repeat the complete sequence of aurally presented items after a tone as correctly as possible. Sequence length was adaptive, adjusting after every second trial of eight trials per condition. If the whole sequence was repeated correctly, the child was awarded with points worth the length of the sequence; an incorrect response was awarded with points worth the sequence length minus 1.

Redintegration was operationalized as the difference between scores of real and pseudowords. This lexicality effect reflects the benefit of real words over pseudowords: real words can be readily reconstructed from long-term memory, whereas pseudowords do not have an entry in the mental lexicon and thus reconstruction cannot be used as effectively (Gathercole et al., 2001; Grube et al., 2008).

#### 2.2.2 Picture naming

The PC-based test on expressive vocabulary in German for primary students aged 6–10 (*Wortschatz- und Wortfindungstest 6–10*, WWT 6–10, Glück, 2011) was conducted in an individual session. It contains 95 pictures belonging to four subscales: objects (“nouns”), activities (“verbs”), antonyms (“adjectives”), and categories. Each picture is verbally prompted and presented for a maximum of 15 s until the child responds. Although the test only returns one aggregate score across all items, the current study analyzed differential item functioning per subscale (i.e., the four classes mentioned above). The retest reliability for the whole test is reported in the manual to be *r*_tt_ = 0.96.

### 2.3 Analyses

Data were prepared, and figures were created in R (RStudio Team, 2016; R Core Team, 2018) using the package eRm (Mair and Hatzinger, 2007) for DIF analyses. Regression analysis for the DT was performed in SPSS Version 25 (IBM Corp, 2017) following the procedure outlined in the electronic supplement in Thomas et al. (2009) and analogously to the original study.

The identification of items with differential item functioning was carried out separately for each of the four subscales (i.e., nouns, verbs, adjectives, and categories). The procedure entailed three steps: (1) estimating a Rasch model across both groups and a global DIF analysis; (2) identifying single DIF items; (3) subsequent global DIF analysis with the resulting reduced subscale.

In the first step using the R-function RM(), a Rasch model over all items of the respective subscale was fitted for both groups and tested globally for subgroup invariance using the Andersen (1973) likelihood ratio test. A significant result means that invariance is not given. In the second step, all items that systematically vary in their item (difficulty) parameters across the groups were identified and excluded via the iterative procedure called by the function stepwiseIt(). In each iteration, the item with the highest discrepancy between the groups was excluded. The discrepancy is measured by the Wald test, which sets the item parameters in relation to their standard errors, allowing it to be interpreted as a *z*-distributed parameter (Koller et al., 2012). After each iteration, a new Rasch model is estimated with the remaining items, until a stop criterion is reached, that is, until none of the remaining items reveal a significant *z*-score. In the third step, the remaining items of the reduced subscale were again tested globally for subgroup invariance, using the Andersen LR-Test and its non-parametric variant, the quasi-exact statistic *T*_10_. In addition, the quasi-exact statistic *T*_11_ tests for local stochastic independence, that is, the one-dimensionality of the subscale.

After removing the DIF items from their respective subscales, a new score was computed for the WWT. As in the original study, a DT analysis was carried out for the redintegration process: The performance in working memory span is predicted by the factors Lexicality, Group, and the reduced WWT score.

## 3 Results

The descriptive results of the full and reduced WWT scores and subscales are shown in Table 2. From the full scales, a total of *k* = 29 items were excluded as subject to DIF, based on their differing item parameters across groups. From subscale “adjectives” only five items had to be excluded, while from subscale “nouns” 12 items were removed.

**Table 2:** Descriptives of WWT (sub)scales: means and standard deviations of the full and reduced WWT 6–10 scores for students with MBID and the TD control group.

	MBID (*n* = 93)		TD (*n* = 114)		Significance
	*M*	*SD*	*M*	*SD*	
Full WWT (*k* = 95)	53.42 (56.3%)	16.79 (17.8%)	54.57 (57.4%)	14.11 (14.9%)	*t*(179.89) = 0.526; *p* = 0.599; *d* = 0.07
Complete subscales					
Nouns (*k* = 26)	14.26 (54.8%)	5.56 (21.4%)	13.75 (52.9%)	4.48 (17.2%)	*t*(175.3) = 0.707; *p* = 0.481; *d* = 0.10
Verbs (*k* = 23)	13.96 (60.7%)	3.90 (17.9%)	14.66 (63.7%)	3.23 (14.0%)	*t*(178.1) = 1.389; *p* = 0.167; *d* = 0.20
Adjectives (*k* = 23)	12.91 (56.1%)	5.04 (21.9%)	13.33 (58.0%)	4.93 (21.4%)	*t*(194.97) = 0.601; *p* = 0.548; *d* = 0.08
Categories (*k* = 23)	12.29 (53.4%)	4.54 (19.7%)	12.82 (55.8%)	4.11 (17.9%)	*t*(187.87) = 0.879; *p* = 0.381; *d* = 0.12
Reduced WWT (*k* = 66)	36.30 (55.0%)	11.75 (17.8%)	38.20 (57.9%)	9.39 (14.2%)	*t*(172.13) = 1.258; *p* = 0.210; *d* = 0.18
Reduced subscales					
Nouns (*k* = 14)	6.73 (48.1%)	3.24 (23.1%)	7.11 (50.8%)	2.41 (17.2%)	*t*(164.06) = 0.950; *p* = 0.344; *d* = 0.14
Verbs (*k* = 17)	10.71 (63.0%)	3.03 (17.9%)	11.18 (65.8%)	2.25 (13.2%)	*t*(165.78) = 1.253; *p* = 0.212; *d* = 0.18
Adjectives (*k* = 18)	9.48 (52.7%)	3.99 (22.1%)	10.36 (57.6%)	4.10 (22.8%)	*t*(198.77) = 1.553; *p* = 0.122; *d* = 0.22
Categories (*k* = 17)	9.40 (55.3%)	3.43 (20.2%)	9.54 (56.1%)	2.95 (17.4%)	*t*(182.61) = 0.324; *p* = 0.746; *d* = 0.05

Upper value represents the sum score of correctly solved items; for easier comparison due to differing numbers of items per subscale, percentages of correctly solved items are provided in parentheses. Due to unequal variances, the corrected degrees of freedom are reported for the *t*-tests.

The results are summarized in Table 3 for each subscale. For the subscale “nouns,” the interpretation is given exemplarily in the following paragraph. All other subscales can be interpreted according to this pattern: First, the number of items and the Andersen LR-Test for the full subscale are shown. The number and a list of excluded items follow in the order of their elimination. For the final reduced subscale, the Andersen LR-Test and the non-parametric *T*_10_ value are reported, in addition to the *T*_11_ value indicating the one-dimensionality of the subscale. A full list of item parameter estimates per group and the results of the Wald test can be obtained in the electronic supplement (Table A1).

**Table 3:** Summary of DIF results per subscale.

Subscale	Items	LR-Test (before)	Excluded items	Items reduced	LR-Test (after)	Non-parametric tests (after)
Nouns	*k* = 26	*LR*(25) = 118.471; *p* < 0.001	bracelet, handrail, crutch, coat, container, heel, ventilator, compass, vending machine, blade, shuttlecock, emblem (*k* = 12)	*k* = 14	*LR*(13) = 17.152; *p* = 0.192	*T*_10_ *p* = 0.082*T*_11_ *p* = 0.032
Verbs	*k* = 23	*LR*(22) = 69.32; *p* < 0.001	to devour, to weigh, to demonstrate, to wait, to bow, to blow (*k* = 6)	*k* = 17	*LR*(16) = 17.424; *p* = 0.359	*T*_10_ *p* = 0.132*T*_11_ *p* = 0.165
Adjectives	*k* = 23	*LR*(22) = 57.057; *p* < 0.001	pointy, smooth, high, ugly, lovely (*k* = 5)	*k* = 18	*LR*(17) = 10.657; *p* = 0.874	*T*_10_ *p* = 0.786*T*_11_ *p* < 0.001
Categories	*k* = 23	*LR*(22) = 67.472; *p* < 0.001	seasons, cosmetics, spices, buildings, plants, construction vehicles (*k* = 6)	*k* = 17	*LR*(16) = 14.872; *p* = 0.534	*T*_10_ *p* = 0.522*T*_11_ *p* < 0.001

“Items” denotes the number of items in the subscale before the exclusion of DIF items, whereas “Items reduced” shows the number of items left in the subscale after the exclusion of DIF items. “LR-Test (before)” refers to the result of the Andersen likelihood ratio test before the exclusion, whereas “LR-Test (after)” shows the result after the exclusion of DIF items. “Excluded items” lists all items that were eliminated in the course of the iterative process, in the order of their elimination. “Non-parametric Tests (after)” provides the *p*-values for the quasi-exact statistics *T*_10_ (indicating subgroup invariance) and *T*_11_ (indicating local stochastic independence), as described in Koller et al. (2012).

### 3.1 Identification of DIF

For the full subscale “nouns” with originally *k* = 26 items, the Andersen LR-Test was significant (*LR*[25] = 118.471; *p* < 0.001). In the course of item elimination, the following 12 items were removed in the given order: *bracelet, handrail, crutch, coat, container, heel, ventilator, compass, vending machine, blade, shuttlecock*, and *emblem*. For the remaining *k* = 14 items in the subscale “nouns,” neither the Andersen LR-Test (*LR*[13] = 17.152; *p* = 0.192) nor the quasi-exact statistic *T*_10_ (*p* = 0.082) showed a significant difference between the groups. However, local stochastic independence (*T*_11_) indicated that also for the reduced subscale, items were still not independent in their probability to be solved (*p* = 0.032). This means that other factors, which were not captured, are likely to influence the solving process of the items. All three other subscales were analyzed in the same procedure, and the corresponding values in Table 3 can be interpreted accordingly.

Summing up, the first research question can be answered that a total of *k* = 29 items, ranging from 5 to 12 items per subscale, had to be excluded as they were likely to cause DIF. The exclusion procedure resulted in a reduced WWT score that was adjusted for DIF consisting of *k* = 66 remaining items. Descriptive statistics for the reduced score (WWT_red_) are contrasted with the original complete score (WWT_full_) in Table 2, shown separately for each group and also for the four subscales. Regarding mean scores, the two groups (MBID and TD) did not differ significantly (*p* = 0.210) in the reduced score, nor in the respective reduced subscales (all *p* > 0.122). As may be expected, the correlation between WWT_red_ and WWT_full_ turned out to be very high with *r* = 0.982 and *p* < 0.001.

### 3.2 Developmental trajectory re-analysis

**Figure 1:**
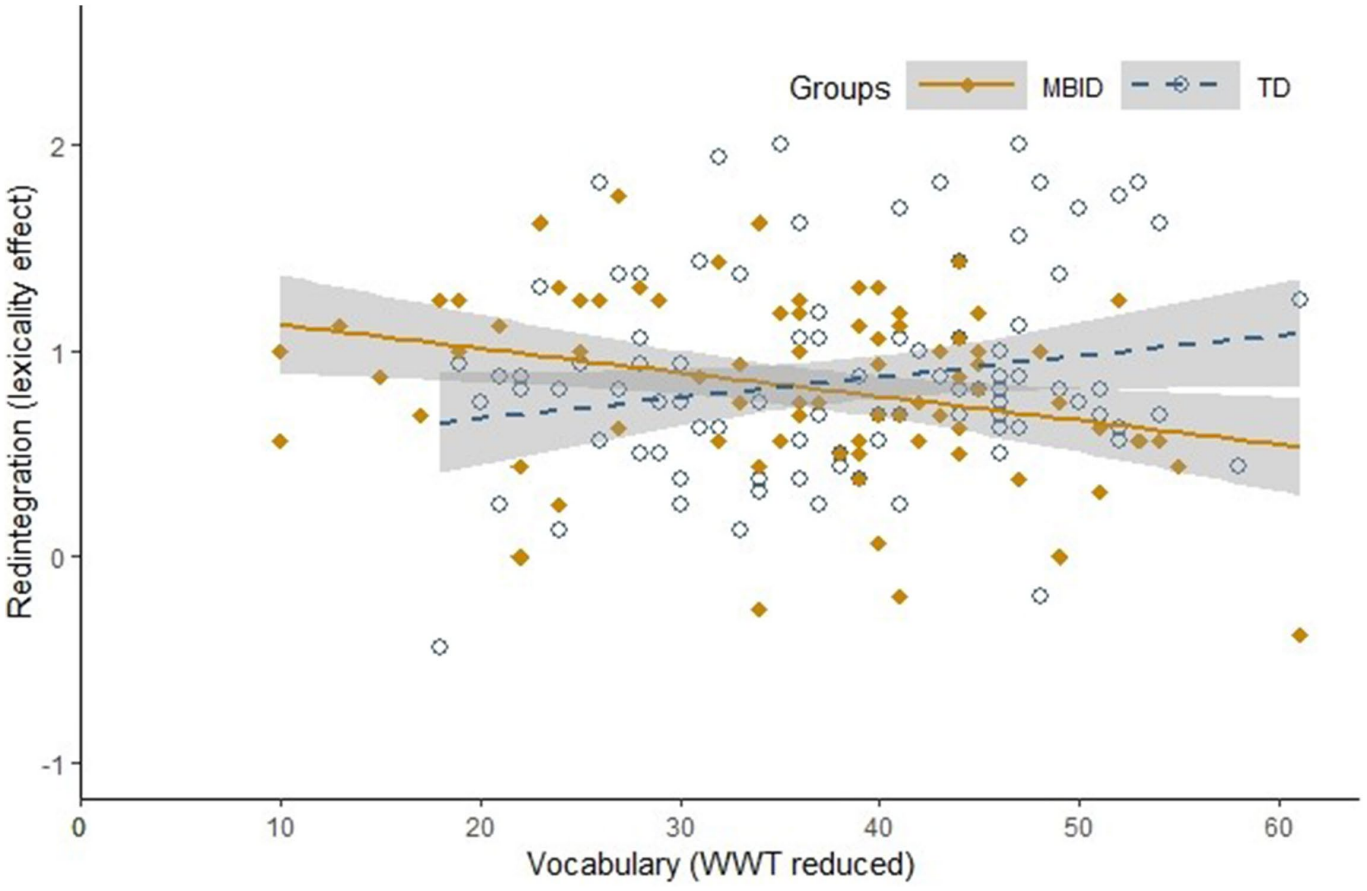
Developmental trajectory of redintegration effectiveness in relation to vocabulary size in students with MBID (filled diamonds) and TD students (circles).

The goal of this study was to re-examine whether students with MBID show differential developmental patterns regarding the effectiveness of redintegration. This answers the question whether the effectiveness of redintegration in students with MBID starts at the same onset level as in TD students, and it can be determined whether developmental progress (in terms of vocabulary size) reflects in a similar way onto redintegration development as in TD students. Because redintegration is operationalized as lexicality effect (i.e., a relative benefit for real words over pseudowords), the task condition Lexicality is included as a predictor in addition to the Group factor and the WWT_red_ score. For easier interpretation, Figure 1 shows the size of the lexicality effect (the difference between real words and pseudowords) in the y-axis, whereas in the regression model, Lexicality is included as a predictor.

Using the DIF-adjusted score WWT_red_ as a predictor, the DT re-analysis on the effectiveness of the redintegration process (i.e., the size of the lexicality effect) yielded a significant difference in intercept, Lexicality × Group; *F*(1, 180) = 5.638; *p* = 0.019; $\eta_{p}^{2}$ = 0.03, implying that the size of the lexicality effect was moderated by the group factor. Parallel to the original study with WWT_full_, this intercept difference favored students with MBID, who show stronger redintegration effectiveness when vocabulary size is low. The difference in slopes, denoted by the triple interaction Lexicality × Group × WWT_red_; *F*(1, 180) = 6.967; *p* = 0.009; $\eta_{p}^{2}$ = 0.039, showed that the groups differed regarding their developmental relations: The relationship between vocabulary size (WWT_red_) and redintegration effectiveness (Lexicality effect) was moderated by the Group factor. As Figure 1 suggests, the redintegration effectiveness of students with MBID decreases with growing vocabulary size. It should be noted that there is one data point to be considered as a potentially influential outlier (see Figure 1, the individual on the far right at the bottom); however, when removing this outlier from the analysis, the results did not substantially change: Lexicality × Group *F*(1, 179) = 4.331; *p* = 0.039; $\eta_{p}^{2}$ = 0.024; and the triple interaction Lexicality × Group × WWT_red_ *F*(1, 179) = 5.067; *p* = 0.025; $\eta_{p}^{2}$ = 0.028.

These results confirm the finding of the original study that vocabulary size interacts with redintegration effectiveness, also when DIF can be excluded as a possible cause. Thus, it appears more plausible to assume a differential developmental pattern in redintegration development for children with MBID. The greater the vocabulary size is in students with MBID, the weaker becomes their redintegration effectiveness.

## 4 Discussion

This study sought to discard DIF as a methodological explanation for the finding of a differential developmental relation between vocabulary size and redintegration effectiveness in students with MBID (Bruns et al., 2019). For this purpose, the items of the WWT were iteratively analyzed using an IRT approach. The WWT turned out to be substantially affected by DIF, as almost one-third of all items (*k* = 29) had to be eliminated due to systematic differences in their difficulty parameters between the groups. However, the main result was not affected by DIF since the differential developmental relation in the DT remained significant with the reduced vocabulary score (*k* = 66). Therefore, DIF can most likely be ruled out as the reason for the difference between the groups, so other more content-related interpretations should be considered. In particular, this means that the pattern of development of the redintegration process in working memory in children with MBID reveals an atypicality that goes beyond a mere delay (Thomas et al., 2009) as it deviates from the pattern expected by mental age.

As limitations should be mentioned that the exclusion process was not theoretically informed but merely data-driven based on the fit values. The subscales did not all appear to be one-dimensional, as the *T*_11_ indicator for local stochastic independence was significant for all but the verb subscales, suggesting that other factors might influence the item difficulty. Furthermore, invariance could only be tested for the WWT but not the redintegration measure, due to its adaptive procedure. A further limitation is the exclusion of items with medium item difficulty parameters, especially for the subscale “nouns.” Finally, it should be highlighted that the data were cross-sectional, so that “development” should not be understood literally; using a broad (mental) age range in a DT approach still allows to detect differential developmental patterns. Regarding the generalizability, it has to remain open whether this finding is specific for MBID or if it could also be observed in children with other forms of learning difficulties, such as specific learning disorders or language impairment.

If redintegration is understood as the process of reconstructing partly degraded working memory traces from knowledge stored in long-term memory (Schweickert, 1993; Roodenrys and Miller, 2008), one should expect that a greater vocabulary should facilitate the reconstruction and hence support redintegration. While this effect was not detectable in typically developing students, it is reliably negatively correlated in students with MBID as a greater mental lexicon seems to have detrimental effects on their use of LTM-knowledge for reconstruction in working memory. Now that DIF can be excluded, the conclusion can be more strongly supported that children with MBID show a structurally different way of using long-term memory for the reconstruction of memoranda in working memory. It is remarkable that the effect holds exclusively for the combination of redintegration effectivity with vocabulary size, while no other developmental indicator (chronological age or cognitive capacity) and neither of the other two dependent variables revealed a similar interaction effect (Bruns et al., 2019). It can be concluded that this interaction poses a singularity in the working memory of children with MBID. Therefore, notions of suboptimal organization of the mental lexicon (Kenett et al., 2016) or a poorer inhibition (Danielsson et al., 2012) of detractors, whose number increases with growing vocabulary size, could become more plausible explanations. This could be translated to a “confusion” hypothesis, suggesting that a larger mental lexicon may lead to more confusion (i.e., more possible distractors), thus hampering the redintegration effect. A better understanding of these cognitive processes and difficulties in students with MBID will ultimately help to tailor interventions more closely to their needs.

## Data Availability

The raw data supporting the conclusions of this article will be made available by the author, without undue reservation.
