# Supplementary material for: Re-analyzing and confirming a differential use of redintegration in students with mild and borderline intellectual disabilities
Source: Front Psychol. 2024 Aug 22;15:1278458. doi: 10.3389/fpsyg.2024.1278458 (PMC11374732; doi:10.3389/fpsyg.2024.1278458)
Supplement: Supplementary file 1 [file Table_1.DOCX]

Table A1

***Parameters per item in the WWT 6-10 and results of the Wald-Test for the group comparison between students with MBID and the TD control group.***

| Subscale | Targetword | Group MBID | | Group TD | | Wald-Test before elimination | | Wald-Test after elimination | |
| --- | --- | --- | --- | --- | --- | --- | --- | --- | --- |
|  |  | Beta | S.E. | Beta | S.E. | z-statistic | p-value | z-statistic | p-value |
| Noun | Schubkarre | 1.287 | 0.282 | 1.837 | 0.274 | -1.398 | .162 | -0.277 | .782 |
| Noun | Hocker | -0.824 | 0.237 | -0.708 | 0.204 | -0.370 | .711 | 0.706 | .480 |
| Noun | Krücke | 1.458 | 0.293 | 0.331 | 0.204 | 3.156 | .002 |  |  |
| Noun | Automat | 1.458 | 0.293 | 0.780 | 0.215 | 1.866 | .062 |  |  |
| Noun | Container | 1.053 | 0.269 | 0.120 | 0.202 | 2.775 | .006 |  |  |
| Noun | Pyramide | 1.371 | 0.287 | 2.398 | 0.331 | -2.346 | .019 | -1.311 | .190 |
| Noun | Federball | 0.979 | 0.265 | 0.417 | 0.206 | 1.673 | .094 |  |  |
| Noun | Kompass | 1.371 | 0.287 | 0.640 | 0.211 | 2.052 | .040 |  |  |
| Noun | Ellenbogen | 1.743 | 0.314 | 1.760 | 0.268 | -0.042 | .966 | 1.065 | .287 |
| Noun | Geländer | -0.371 | 0.235 | 0.640 | 0.211 | -3.203 | .001 |  |  |
| Noun | Satellit | -1.237 | 0.245 | -1.399 | 0.223 | 0.490 | .624 | 1.448 | .147 |
| Noun | Orchester | -1.830 | 0.267 | -1.978 | 0.254 | 0.402 | .688 | 1.199 | .231 |
| Noun | Knospe | -1.116 | 0.242 | -0.498 | 0.201 | -1.962 | .050 | -0.928 | .353 |
| Noun | Ferse | 0.445 | 0.246 | 0.331 | 0.204 | 0.358 | .720 | 1.564 | .118 |
| Noun | Mantel | 1.053 | 0.269 | 0.079 | 0.201 | 2.900 | .004 |  |  |
| Noun | Ventilator | 2.347 | 0.374 | 1.302 | 0.238 | 2.357 | .018 |  |  |
| Noun | Schleier | -1.488 | 0.253 | -0.837 | 0.206 | -1.995 | .046 | -1.045 | .296 |
| Noun | Riegel | -2.220 | 0.289 | -1.451 | 0.225 | -2.099 | .036 | -1.326 | .185 |
| Noun | Fackel | -0.427 | 0.235 | -0.623 | 0.203 | 0.632 | .528 | 1.768 | .077 |
| Noun | Wappen | -1.116 | 0.242 | -1.503 | 0.228 | 1.166 | .244 |  |  |
| Noun | Klinge | -0.427 | 0.235 | -1.015 | 0.210 | 1.865 | .062 |  |  |
| Noun | Absatz | -0.314 | 0.235 | -1.107 | 0.213 | 2.499 | .012 |  |  |
| Noun | Armreif | -1.830 | 0.267 | -0.086 | 0.200 | -5.226 | < .001 |  |  |
| Noun | Schnalle | -1.978 | 0.274 | -1.348 | 0.221 | -1.788 | .074 | -0.961 | .337 |
| Noun | Henkel | -1.237 | 0.245 | -0.881 | 0.207 | -1.108 | .268 | -0.112 | .911 |
| Noun | Gipfel | 1.848 | 0.323 | 2.799 | 0.386 | -1.889 | .059 | -0.929 | .353 |
| Verben | fressen | -1.467 | 0.241 | -0.249 | 0.202 | -3.874 | < .001 |  |  |
| Verben | zeigen | 2.664 | 0.478 | 2.161 | 0.382 | 0.823 | .410 | 1.075 | .282 |
| Verben | entgleisen | -3.368 | 0.377 | -2.807 | 0.278 | -1.198 | .231 | -1.546 | .123 |
| Verben | jonglieren | 0.791 | 0.264 | 1.067 | 0.259 | -0.748 | .455 | -0.537 | .592 |
| Verben | schälen | 2.253 | 0.409 | 1.765 | 0.327 | 0.933 | .351 | 1.173 | .241 |
| Verben | schieben | 1.097 | 0.283 | 0.808 | 0.241 | 0.778 | .437 | 0.991 | .322 |
| Verben | balancieren | 1.683 | 0.335 | 1.765 | 0.327 | -0.175 | .861 | 0.052 | .958 |
| Verben | warten | 1.939 | 0.365 | 0.869 | 0.245 | 2.433 | .015 |  |  |
| Verben | demonstrieren | -1.526 | 0.243 | -2.518 | 0.256 | 2.805 | .005 |  |  |
| Verben | abtrocknen | -0.545 | 0.228 | -1.011 | 0.200 | 1.535 | .125 | 1.54 | .123 |
| Verben | abbrechen | -0.335 | 0.229 | 0.094 | 0.209 | -1.382 | .167 | -1.257 | .209 |
| Verben | verblühen | -1.970 | 0.261 | -1.559 | 0.211 | -1.224 | .221 | -1.373 | .170 |
| Verben | wehen | -1.647 | 0.247 | -0.890 | 0.199 | -2.385 | .017 |  |  |
| Verben | wiegen | 1.570 | 0.323 | 0.231 | 0.214 | 3.452 | .001 |  |  |
| Verben | dirigieren | -2.775 | 0.313 | -2.586 | 0.261 | -0.465 | .642 | -0.772 | .44 |
| Verben | trösten | 0.395 | 0.246 | 0.326 | 0.217 | 0.210 | .834 | 0.382 | .703 |
| Verben | verbeugen | -0.755 | 0.229 | 0.094 | 0.209 | -2.738 | .006 |  |  |
| Verben | reiben | -1.352 | 0.238 | -1.427 | 0.207 | 0.238 | .812 | 0.154 | .877 |
| Verben | ziehen | 1.016 | 0.278 | 1.462 | 0.293 | -1.104 | .270 | -0.888 | .374 |
| Verben | schimpfen | 0.457 | 0.248 | 0.139 | 0.211 | 0.977 | .328 | 1.138 | .255 |
| Verben | abschleppen | 0.720 | 0.260 | 0.581 | 0.228 | 0.403 | .687 | 0.598 | .550 |
| Verben | stricken | -0.650 | 0.228 | -0.081 | 0.205 | -1.856 | .063 | -1.763 | .078 |
| Verben | brüllen | 1.805 | 0.349 | 1.765 | 0.327 | 0.085 | .932 | 0.315 | .753 |
| Adjective | alt | 2.425 | 0.411 | 2.482 | 0.355 | -0.105 | .916 | 0.069 | .945 |
| Adjective | lieb | 1.965 | 0.361 | 3.570 | 0.514 | -2.554 | .011 |  |  |
| Adjective | früh | 2.425 | 0.411 | 2.131 | 0.323 | 0.564 | .573 | 0.753 | .452 |
| Adjective | weich | 2.259 | 0.392 | 2.764 | 0.387 | -0.918 | .358 | -0.762 | .446 |
| Adjective | wild | -3.641 | 0.431 | -3.327 | 0.319 | -0.585 | .558 | -0.222 | .824 |
| Adjective | wolkenlos | -1.670 | 0.253 | -1.656 | 0.229 | -0.042 | .967 | 0.525 | .600 |
| Adjective | einfach | -0.239 | 0.237 | -0.160 | 0.226 | -0.242 | .809 | 0.271 | .786 |
| Adjective | glatt | -0.795 | 0.234 | -2.033 | 0.239 | 3.698 | < .001 |  |  |
| Adjective | einfarbig | -1.606 | 0.251 | -1.656 | 0.229 | 0.146 | .884 | 0.715 | .475 |
| Adjective | mutig | -1.130 | 0.238 | -1.502 | 0.226 | 1.133 | .257 | 1.714 | .087 |
| Adjective | vertraut | -1.870 | 0.262 | -1.656 | 0.229 | -0.615 | .538 | -0.058 | .954 |
| Adjective | traurig | -0.239 | 0.237 | -0.056 | 0.227 | -0.558 | .577 | -0.054 | .957 |
| Adjective | nah | 0.112 | 0.245 | 0.920 | 0.252 | -2.301 | .021 | -1.91 | .056 |
| Adjective | langweilig | -0.575 | 0.234 | -0.263 | 0.224 | -0.961 | .336 | -0.437 | .662 |
| Adjective | hoch | 1.022 | 0.285 | 0.157 | 0.231 | 2.363 | .018 |  |  |
| Adjective | innen | 0.709 | 0.267 | 1.121 | 0.260 | -1.105 | .269 | -0.766 | .444 |
| Adjective | hässlich | 2.106 | 0.375 | 1.053 | 0.257 | 2.316 | .021 |  |  |
| Adjective | sauer | -0.630 | 0.234 | -0.761 | 0.221 | 0.409 | .682 | 0.969 | .332 |
| Adjective | unzufrieden | -0.630 | 0.234 | -0.465 | 0.223 | -0.511 | .609 | 0.029 | .977 |
| Adjective | spitz | -0.352 | 0.236 | -1.604 | 0.228 | 3.818 | < .001 |  |  |
| Adjective | altmodisch | -0.296 | 0.236 | -0.314 | 0.224 | 0.054 | .957 | 0.581 | .562 |
| Adjective | trocken | 1.835 | 0.348 | 2.358 | 0.343 | -1.070 | .285 | -0.895 | .371 |
| Adjective | ungefährlich | -1.187 | 0.239 | -1.104 | 0.222 | -0.253 | .800 | 0.318 | .751 |
| Categorie | Gemüse | 1.826 | 0.320 | 1.413 | 0.253 | 1.010 | .313 | 0.842 | .400 |
| Categorie | Getreide | -1.180 | 0.239 | -1.513 | 0.221 | 1.025 | .306 | 0.818 | .413 |
| Categorie | Pflanzen | -1.123 | 0.237 | -0.329 | 0.201 | -2.557 | .011 |  |  |
| Categorie | Kosmetika | 2.764 | 0.413 | 1.163 | 0.238 | 3.362 | .001 |  |  |
| Categorie | Gepäck | -1.066 | 0.236 | -0.915 | 0.205 | -0.484 | .629 | -0.672 | .502 |
| Categorie | Gewürze | 0.552 | 0.248 | -0.453 | 0.201 | 3.148 | .002 |  |  |
| Categorie | Instrumente | 2.445 | 0.377 | 1.626 | 0.268 | 1.770 | .077 | 1.603 | .109 |
| Categorie | Kopfbedeckungen | -2.033 | 0.277 | -1.722 | 0.230 | -0.866 | .387 | -1.094 | .274 |
| Categorie | Geschirr | 0.748 | 0.256 | 0.832 | 0.223 | -0.247 | .805 | -0.407 | .684 |
| Categorie | Sportarten | 1.267 | 0.282 | 0.731 | 0.219 | 1.502 | .133 | 1.338 | .181 |
| Categorie | Küchengeräte | -1.010 | 0.235 | -1.002 | 0.207 | -0.027 | .979 | -0.218 | .827 |
| Categorie | Feste | -1.180 | 0.239 | -0.703 | 0.202 | -1.526 | .127 | -1.705 | .088 |
| Categorie | Schmuck | 2.304 | 0.363 | 2.674 | 0.386 | -0.698 | .485 | -0.872 | .383 |
| Categorie | Werkzeuge | 2.445 | 0.377 | 1.960 | 0.297 | 1.011 | .312 | 0.838 | .402 |
| Categorie | Besteck | 0.682 | 0.253 | 0.682 | 0.218 | 0.001 | .999 | -0.16 | .873 |
| Categorie | Baufahrzeuge | -2.374 | 0.302 | -1.513 | 0.221 | -2.300 | .021 |  |  |
| Categorie | Lebensmittel | -0.737 | 0.231 | -1.002 | 0.207 | 0.855 | .392 | 0.663 | .507 |
| Categorie | Jahreszeiten | -0.200 | 0.231 | 0.992 | 0.230 | -3.656 | < .001 |  |  |
| Categorie | Insekten | -0.845 | 0.232 | -0.745 | 0.203 | -0.327 | .744 | -0.509 | .611 |
| Categorie | Möbel | 0.367 | 0.242 | 0.006 | 0.203 | 1.143 | .253 | 0.975 | .330 |
| Categorie | Gebäude | -1.417 | 0.246 | -0.536 | 0.201 | -2.775 | .006 |  |  |
| Categorie | Zeichen | -0.630 | 0.230 | -0.329 | 0.201 | -0.986 | .324 | -1.153 | .249 |
| Categorie | Berufe | -1.607 | 0.254 | -1.319 | 0.214 | -0.867 | .386 | -1.073 | .283 |
| *Notes*. Beta: Item parameter of the Rasch model that was estimated separately for each group. The values for Beta and S.E. refer to the Rasch model with all items (i.e., before item elimination). The reduced version after elimination leads to different item parameters which are omitted here. Instead, the results of the Wald test after elimination are shown in the last two columns. For items that were excluded, no values are shown in the Wald test. | | | | | | | | | |
